# Spontaneous uterine rupture and surgical repair at 21 weeks gestation with progression to live birth: a case report

**DOI:** 10.1186/s12884-018-1761-x

**Published:** 2018-05-04

**Authors:** Lesley Hawkins, Deborah Robertson, Helena Frecker, Howard Berger, Abheha Satkunaratnam

**Affiliations:** 1 Department of Obstetrics and Gynaecology, University of Toronto, 123 Edward St, 12th Floor, Toronto, ON M5G1E2 Canada; 2 Department of Obstetrics and Gynaecology, St Michael’s Hospital, 308-55 Queen St East, Toronto, ON M5C1R6 Canada; 3 Department of Obstetrics and Gynaecology, Michael Garron Hospital, Suite 311, 658 Danforth Avenue, M4J5B9 Toronto, Ontario Canada

**Keywords:** Uterine rupture, Spontaneous uterine rupture, Uterine wall defect, Second trimester

## Abstract

**Background:**

Uterine rupture in the non-laboring uterus is a rare occurrence, which can lead to significant morbidity and mortality for the mother and fetus. Management of this presentation is complex at pre-viable gestations.

**Case presentation:**

A 35 year old primigravid woman with multiple previous myomectomies presented with spontaneous complete thickness uterine rupture at 21 weeks gestation. A 10 cm myometrial defect and iatrogenic amniotomy were surgically corrected with fetal preservation. This led to pregnancy continuation to 32 weeks gestation when elective cesarean delivery resulted in excellent neonatal outcome.

**Conclusions:**

Early surgical diagnosis, multidisciplinary team approach, iatrogenic amniotomy and continuous two-layer myometrial closure were factors that contributed to pregnancy prolongation in this large myometrial rupture.

## Background

Uterine rupture is defined as complete separation of the myometrium [1, 2]. It can occur in the laboring or non-laboring uterus, the latter known as spontaneous uterine rupture. Spontaneous uterine rupture is a rare occurrence which can lead to maternal hemorrhage, placental abruption and extrusion of the amniotic sac and fetal parts through the uterine defect. This can result in significant consequences for both the mother and fetus, including blood transfusion, hysterectomy, urologic injury, neonatal respiratory distress, perinatal asphyxia and maternal or fetal death [3–5]. A history of uterine surgery has been identified as the most common risk factor for spontaneous uterine rupture in a small cohort of cases [6]. Less common causes include iatrogenic uterine perforation, invasive placenta, congenital anomalies, trauma and sacculation of the entrapped retroverted uterus [7].

Management of this rare pregnancy complication requires several considerations. Cesarean delivery with either uterine repair or hysterectomy may be appropriate at fetal viability. However, when the fetus is previable or extremely premature, management decisions are complex. Termination of the pregnancy with uterine repair or hysterectomy was the traditional approach [8]. In recent years, repair of uterine rupture in the second and early third trimesters has been reported, with successful delay of delivery [9–11]. We describe a rare case of spontaneous uterine rupture in the mid-second trimester and successful surgical repair including inadvertent iatrogenic amniotomy with continuation of pregnancy to 32 weeks gestation.

## Case presentation

A 35-year old primigravid woman presented to hospital at 21 weeks 2 days gestation. She described a two-day history of periumbilical abdominal discomfort that suddenly became severe, waking her from sleep. There was no history of trauma. She was afebrile and had no other gastrointestinal symptoms. She had previously undergone four hysteroscopic myomectomies and one open myomectomy, during which 5 uterine incisions were made, including a fundal incision, and over 100 fibroids were resected. The indication for these surgeries was primary infertility, abnormal uterine bleeding and anemia. The interval between open myomectomy and conception was 26 months. The current pregnancy was spontaneously conceived and had been progressing normally.

At presentation, blood pressure was 99/54, heart rate 84 beats per minute, respiratory rate 20, temperature 36 degrees Celsius (96.8 degrees Farenheit) and fetal heart rate 160 beats per minute. Abdominal examination revealed diffuse peritonitis and significant tenderness at the uterine fundus. Hemoglobin at presentation was 87 g/L and white blood cell count was 10.89 E9/L (reference range 120-160 g/L and 4.00 to 11.00 E9/L, respectively). Ultrasound examination showed a live singleton fetus with normal amniotic fluid volume. The placental position was left posterofundal. Moderate volume hemoperitoneum was seen. Ovaries appeared normal bilaterally; the appendix was not visualized. Evaluation of the uterine wall demonstrated no focal uterine disruption or thinning, but was limited due to patient tenderness and bowel gas, therefore, a Magnetic Resonance Imaging (MRI) study was recommended. Urgent unenhanced abdominal MRI, completed that evening, 6 h following ultrasound examination, showed a sentinel clot at the left aspect of the uterine fundus, where the myometrium was markedly thinned, suspected to be the source of hemorrhage. During the diagnostic work-up, vital signs were stable and hemoglobin reached a stable nadir of 71 g/L.

Differential diagnosis included fibroid degeneration or torsion, placenta percreta, concealed placental abruption, uterine rupture and non-obstetrical causes. A patient care conference and complex consent process was undertaken to include several potential scenarios and outcomes. The patient was counselled extensively around possible complications of surgical repair, including prolongation of the pregnancy leading to severe prematurity, preterm rupture of membranes, and risks of unsuccessful repair, including hysterectomy and fetal loss. With a demonstration of an understanding of these risks, she consented to a diagnostic laparoscopy, uterine repair with possible laparotomy, possible hysterotomy and evacuation of the fetus and possible hysterectomy.

**Fig. 1:**
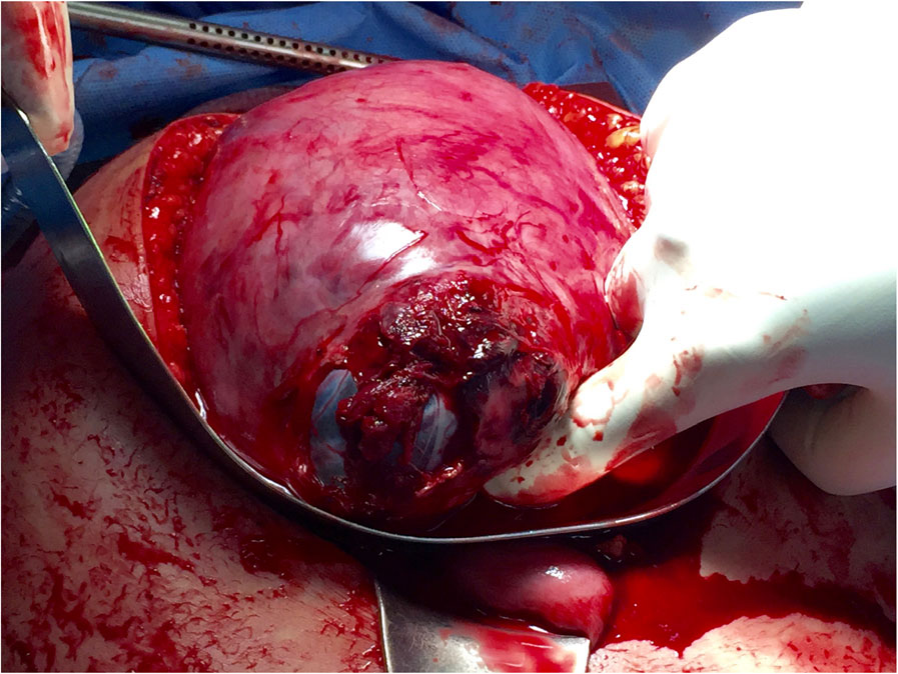
At laparotomy, the 10 cm fundal complete uterine wall defect with protruding chorioamniotic membrane

**Fig. 2:**
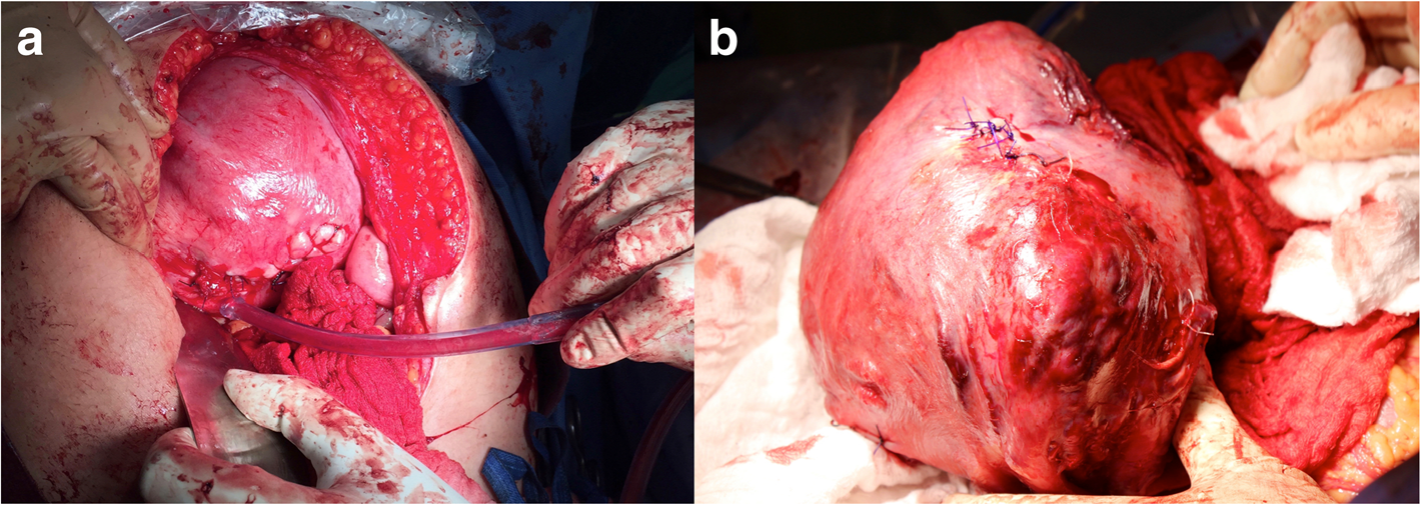
**a** Two-layer closure of uterine defect following iatrogenic amniotomy and repair with surgical clips (not shown). **b**. Cesarean delivery: previous left fundal uterine rupture repair site intact, with remaining polydioxanone suture visible

The following day, on the second day of admission, at 21 weeks 3 days gestation, the patient was taken to the operating room. The procedure was initiated laparoscopically via a left upper quadrant veress needle entry. A 10 cm complete thickness uterine fundus rupture was diagnosed (Fig. 1). Active bleeding was visualized from the edge of the rupture, and the chorioamniotic membranes were visibly bulging through the defect. Large hemoperitoneum (estimated 1000 mL) was present. Given the extent and location of the defect, the size of the gravid uterus and visualization laparoscopically, we converted to midline laparotomy. Closure of the defect was then undertaken. Unintentional iatrogenic amniotomy occurred due to the suture needle shaft scratching the membranes. This resulted in two adjacent 2 mm defects in the amniotic sac and partial loss of amniotic fluid volume. Two large vascular clips were applied to each defect for repair. We introduced a 22 French foley catheter into the myometrial defect and inflated the balloon to displace the amniotic sac to facilitate uterine repair. With the membranes reduced, a two-layer uterine closure was successfully completed using full thickness continuous 0 polyglactin 910 (Vicryl) and imbricating 2–0 Polydioxanone (PDS, Ethicon, USA) sutures, respectively (Fig. 2). EVICEL and SURGICEL SnoW (Ethicon, USA) were applied to the area of repair to affect hemostasis. Intraoperatively, the patient required transfusion of 7 units packed erythrocytes, 2 units fresh frozen plasma and 1 unit cryoprecipitate; estimated total blood loss was 2500 mL.

**Fig. 3:**
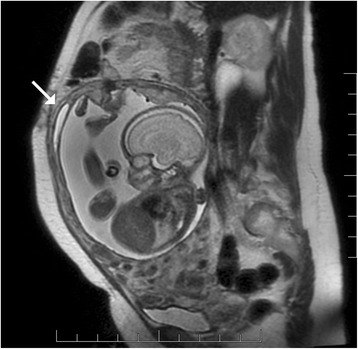
MRI showing 4 mm fundal uterine wall thickness and 0.7 cm by 5 cm amniotic fluid leak (arrow)

The patient’s postoperative course was uncomplicated. Indomethacin was administered for tocolysis per the standard of care at our centre (100 mg per rectum then 25 mg per os every 6 h for 4 doses). Postoperative intravenous cefazolin was administered for 24 h. Fetal surveillance demonstrated amniotic fluid re-accumulation, appropriate growth parameters (estimated fetal weight 60–85%ile) and normal fetal well-being. Amniotic fluid index (AFI) was 6.5 cm on postoperative day (POD) 3, 9 cm on POD4, 8.9 cm on POD5 and 12.5 cm on POD12. Myometrial thickness at the repair site was evaluated at several time points by ultrasound and ranged from 0.29 to 0.4 cm. Ultrasound and MRI at 27 weeks gestation showed a 0.7 cm by 5 cm area of chorion-amnion separation at the site of repair which remained stable throughout our patient’s course (Fig. 3). The patient was transferred to a centre with Level 3 neonatal care at 24 weeks gestation, for continued inpatient observation, then transferred back to our Level 2.5 centre at 30 weeks gestation. A course of antenatal corticosteroids was administered at 25 weeks gestation.

Using a multidisciplinary approach, a second patient care conference was held involving Obstetrical, Gynecologic, Maternal Fetal Medicine, Pediatric, Anesthesia and Nursing teams to plan delivery. Consensus decision was achieved for planned cesarean delivery at 32 weeks 1 day gestation. This gestation was felt to be an optimal compromise between fetal maturity, and the maternal and fetal risks of sudden uterine rupture. A liveborn female, weighing 1884 g with Apgars of 8 and 9 at 1 and 5 min, respectively, was transferred to the Neonatal Intensive Care Unit in good condition (umbilical artery pH 7.28). Intraoperatively, the previous uterine wall repair site was found to be intact at the fundus (Fig. 2B). Significant neovascularization was found on the serosal surface of the uterus. The myometrium had limited integrity, with several thin and weak areas. The fundal posterior placenta was markedly adherent; approximately 95% of it was felt to be removed. Estimated blood loss was 2000cm^3^. Three surgical clips were retrieved intraoperatively, but the fourth was not easily palpated and given the clinical circumstances, left behind. In the perioperative period, the patient received 3 units packed erythrocytes.

Mother and infant were stable postpartum. The patient was discharged home on POD6. Her infant daughter received continuous positive airway pressure (CPAP) until day 2 of life. During her neonatal course, she underwent phototherapy for neonatal jaundice and was diagnosed with suspected cow’s milk protein allergy. She met appropriate milestones and was discharged home on day 36 of life. Mother passed the last surgical clip spontaneously per vagina 10 weeks postpartum.

## Discussion and conclusions

Rupture of the pregnant uterus at any time in gestation has a prevalence of 0.05% in population-based studies [4]. Spontaneous antenatal uterine rupture, however, has seldom been reported. A rare event, ten cases of spontaneous uterine rupture in the second trimester with successful repair have been described in a recent review by Surico et al. (2016). These cases presented at 13 to 26 weeks gestation and had a median pregnancy prolongation interval of 12 weeks. Identified risk factors in that series included previous cesarean delivery and previous uterine surgery, although some cases presented with no apparent risk factor [11].

Accurate pre-operative diagnosis was a challenge in this case. The most common presenting symptom of spontaneous rupture is sudden onset of severe abdominal pain, which happened to our patient. Vaginal bleeding, shock and fever have also been described [11]. Although ultrasound examination confirmed hemoperitoneum, its utility in diagnosing uterine rupture was limited. MRI has been shown to be useful in the work-up of acute abdomen in pregnancy in hemodynamically stable patients and has demonstrated superior accuracy in evaluation of uterine wall defects [12, 13].

A variety of repair techniques have been used in cases of spontaneous uterine rupture, including polyglactin 910, PDS, Monocryl, chromic catgut sutures in interrupted and running fashion, GoreTex and Tachocomb patches and Vicryl and Surgicel mesh, which all resulted in successful pregnancy prolongation [11]. In our experience, the protruding amniotic sac provided significant tension to the edges of the ruptured myometrium, posing a technical challenge in safely and effectively opposing the edges. The iatrogenic amniotic sac defects were repaired with large vascular clips, providing an effective seal. In open fetal procedures such as in myelomeningocele, sacrococcygeal teratoma and fetal lung lesions, techniques used during creation of the hysterotomy include absorbable polyglycolic acid stapling devices and continuous locked running sutures which oppose the uterine wall edge to the chorioamniotic membranes, thus facilitating a water-tight hysterotomy closure at the end of the procedure [14–17]. In some cases of spontaneous uterine rupture, intraoperative needle amniocentesis has also been described [18–20]. In this case, unintentional amniotomy was found to be beneficial. However, planned amniotomy could be considered in future cases to facilitate closure. Amnioinfusion was not carried out intraoperatively, as we felt that myometrial healing of this compromised area would be better achieved under reduced tension. Serial postoperative ultrasounds showed complete reaccumulation of amniotic fluid several days postoperatively and maintenance of normal amniotic fluid index until delivery. Therefore, we have identified that iatrogenic amniotomy can be successfully repaired with surgical clips in sterile conditions; this is the first known description of its kind.

Several unique maternal and fetal concerns arise following emergency fetal surgery in addition to chorioamniotic separation and oligohydramnios. These include risk of repair dehiscence, postoperative infection, premature rupture of membranes and preterm labor [14]. Serial imaging was used to evaluate the uterine wall thickness post-repair (range 0.29 to 0.4 cm). A cohort of normal pregnancies demonstrated mean fundal myometrial thickness in the second trimester of 5.9mm [21], but the utility of scar monitoring in this unique circumstance is unknown and cannot be compared to surveillance protocols used to plan trial of labor after cesarean. Postoperative antibiotics were given for 24 h. Alternative regimens include intrauterine antibiotics infused in warmed Lactated Ringer’s solution, in addition to systemic antibiotics [14]. Our patient did not have any symptoms or signs of preterm labor throughout her course. Various protocols for tocolysis following fetal surgery have been described [14, 22, 23], and could be considered in uterine rupture cases.

A multidisciplinary team was formed to plan delivery including the patient’s wishes. Factors that contributed to the decision-making process included fetal maturity, risk of developmental sequelae of severe prematurity, risk of secondary uterine rupture, risk of premature labor, and associated risk of fetal demise. Planned cesarean delivery was predicted to provide significantly superior maternal and neonatal outcomes as compared to expectant management with emergency delivery, due to the risk of secondary uterine rupture and its consequences. Confirmation of fetal pulmonary maturity has been reported in a similar case [10], but was not performed as it was decided that the results would not influence the timing of delivery. Risks of subsequent pregnancy and recurrent uterine rupture were discussed extensively; the patient declined the options of concurrent hysterectomy or tubal interruption. The same surgical team that was involved in the uterine rupture repair carried out the cesarean delivery in order to provide continuity of clinical care and surgical expertise.

In conclusion, a multidisciplinary team approach was crucial to navigating medical and ethical considerations. Insights into successful technical closure of uterine wall dehiscence included iatrogenic amniotomy and its repair and foley catheter balloon reduction of amniotic membranes allowing for superior myometrial reapproximation. Furthermore, these novel techniques resulted in successful continuation of pregnancy with excellent neonatal outcome after this rare presentation of pre-viable spontaneous uterine rupture.

## Abbreviations

AFI: Amniotic fluid index
CPAP: Continuous positive airway pressure
MRI: Magnetic resonance imaging
PDS: Polydioxanone
POD: Postoperative day
